# A Comparison of Nerve Injury in Cross Versus Lateral Pinning Fixation of Displaced Supracondylar Humerus Fracture

**DOI:** 10.7759/cureus.70404

**Published:** 2024-09-28

**Authors:** Muhammad Mannan, Shahzeen Eisha, Asif Afridi

**Affiliations:** 1 Orthopedic Surgery, University Hospital Birmingham, Birmingham, GBR; 2 Orthopedics and Trauma, Sheikh Zayed Medical College, Rahim Yar Khan, PAK; 3 Trauma and Orthopedics, Royal College of Surgeons in Ireland, Brighton, GBR; 4 Orthopedic Surgery, Sheikh Zayed Medical College, Rahim Yar Khan, PAK; 5 Trauma and Orthopedics, Hayatabad Medical Complex Peshawar, Peshawar, PAK; 6 Trauma and Orthopedics, Queen Elizabeth Hospital Birmingham, Birmingham, GBR

**Keywords:** cross pinning, flynn's criteria, gartland type iii fractures, lateral pinning, supracondylar fracture of the humerus (scfh)

## Abstract

Background and objective: Supracondylar fracture of the humerus (SCFH) is a common pediatric fracture encountered in orthopedic surgery. The most frequently used pinning methods include cross pinning or lateral pinning with two or three pins. However, complications such as ulnar nerve injury can occur, particularly during medial pinning, which necessitates careful isolation of the ulnar nerve and expert surgical intervention. The objective of this study is to compare nerve injury in cross versus lateral pinning fixation of the SCFH.

Materials and methods: The observational study was conducted at Sheikh Zayed Hospital, Rahim Yar Khan, PAK. Patients in group L (n=55) underwent lateral pinning, while those in group C (n=55) received cross pinning. The patients were followed retrospectively postoperatively until radiological union was achieved. The outcomes were assessed using the Flynn criteria, and nerve injury was evaluated in both groups. The study aimed to compare the postoperative results between the two groups based on the specified criteria.

Results: The mean age was 7.28±2.03 years in group L and 8.20±2.21 years in group C. The majority of patients in both groups were male. The left side was more commonly involved, and among the 110 patients enrolled, most sustained the injury while playing. The mean follow-up period was 21.3±1.4 months for group L and 23.5±0.2 weeks for group C. Nerve injuries were reported in six (5.45%) patients in group C, while no nerve injuries were reported in group L.

Conclusion: For type III SCFH, lateral pinning fixation has demonstrated itself to be an effective alternative, yielding excellent functional outcomes. This method not only minimizes the risk of iatrogenic nerve injury, particularly to the ulnar nerve but also provides sufficient stability for the fracture. The results from this study suggest that lateral pinning is a reliable option for treating unstable supracondylar fractures, with a favorable safety profile and outcomes comparable to, if not better than, those achieved with cross pinning. This makes it a valuable technique, especially in settings where the risk of nerve injury is a significant concern.

## Introduction

Supracondylar fracture of the humerus (SCFH) are common pediatric fractures encountered in orthopedic surgery, with approximately 85% occurring in children between the ages of four and 11 [1]. It accounts for 50% to 70% of all elbow fractures in children during the first decade of life [2]. These fractures are traditionally classified into extension or flexion injuries, with the extension type being the most prevalent [3]. The Gartland classification is widely used to categorize pediatric supracondylar fractures. While most type I fractures are managed non-surgically, type II and nearly all type III fractures typically require surgical intervention [4]. Closed reduction with percutaneous pinning is the procedure of choice for displaced Gartland type II, III, and IV fractures [5].

The Kirschner wire (K-wire) can be configured in various ways to stabilize the displaced fracture, with cross pinning and lateral pinning being common configurations. Over the past decade, cross pinning, where pins are inserted medially and laterally through the corresponding epicondyles, has been the most frequently used technique [6]. Although cross or lateral pinning with two or three pins is the most common pinning configuration for SCFH, there is ongoing debate about which approach yields the best functional outcomes, with many studies comparing the two in terms of surgical results [7]. The primary considerations when comparing these procedures are elbow stability and the potential risk of iatrogenic ulnar nerve injury. It has been demonstrated that medial/lateral pinning offers greater mechanical stability compared to lateral pinning alone [8].

A significant concern with cross pinning is the potential for ulnar nerve injury, which necessitates careful ulnar nerve isolation during the procedure [9]. Additionally, ulnar nerve isolation often requires a skin incision, which may be considered an aesthetic disadvantage. To address this issue, closed reduction with lateral pinning has been used more recently; however, it is argued that lateral pinning may result in a loss of reduction compared to cross pinning [10]. There remains a lack of consensus on which pinning method minimizes these risks while providing better functional outcomes.

Neurological complications, particularly ulnar and radial nerve injuries, are significant concerns during the surgical management of SCFH. Ulnar nerve palsy is more commonly associated with medial pinning, with reported incidences ranging from 1.4% to 6.8% depending on the technique used. Radial nerve injury, although less frequent, is most often observed in cases of flexion-type fractures, with reported incidences ranging from 2% to 4% in the literature [11]. However, the concern in surgical settings is primarily iatrogenic nerve injury, which can occur during pin placement. Notably, studies have shown that radial nerve injury due to pinning is very rare and tends to occur more frequently in cross pinning techniques compared to lateral pinning [12]. A recent study reported an ulnar nerve injury rate of 6.8% with cross pinning compared to 0% with lateral pinning [5]. Therefore, this study aims to compare the risk of both ulnar and radial nerve injuries between cross pinning and lateral pinning fixation techniques for SCFH.

## Materials and methods

Study design

This is an observational study where patients were followed up retrospectively after surgery to compare the incidence of ulnar and radial nerve injuries in cross pinning versus lateral pinning fixation for SCFH in pediatric patients. It aims to evaluate the functional outcomes and complications associated with each pinning method, with a focus on determining which technique provides better nerve safety and overall fracture stability. The study was conducted at the Department of Orthopedic Surgery, Sheikh Zayed Medical College and Hospital, Rahim Yar Khan, PAK. The study period was one year (January 1, 2019 to December 31, 2019), with data collection occurring over six months. A total of 110 cases were included in the study; the sample size was calculated using the WHO sample size calculator with a 95% confidence level, a 5% margin of error, and based on reported nerve injury rates of 6.8% for cross pinning and 0% for lateral pinning. Table 1 describes the inclusion and exclusion criteria for the study, providing a clear overview of the patient selection process.

**Table 1:** Inclusion and exclusion criteria for the study SCFH: Supracondylar fracture of the humerus

Criteria	Details
Inclusion criteria	Pediatric patients up to 13 years of age with type III A & B closed extension SCFH
Exclusion criteria	Patients with type I and II fractures, flexion-related injuries, complex fractures, patients aged >13

Methodology

The patients were retrospectively followed postoperatively and were categorized into two groups based on the type of pinning they received. Group C (n=55) consisted of patients who underwent cross pinning, while group L (n=55) included those who had lateral pinning. Each fracture was classified according to the Gartland classification system (Table 2).

**Table 2:** Gartland classification

Type	Description	Displacement	Management
I	Nondisplaced supracondylar fracture	No displacement	Conservative (e.g., casting)
II	Displaced supracondylar fracture with intact posterior cortex	Angulated, but posterior cortex remains intact	Closed reduction and casting
III	Completely displaced supracondylar fracture with no cortical contact	Complete displacement (either posterior or anterior)	Surgical treatment (usually pinning)
IV	Periosteal disruption with instability in both flexion and extension (multidirectional)	Unstable in all planes	Surgical intervention required

Patients were followed up regularly until the radiological union was confirmed. Outcomes were assessed using the Flynn criteria, which evaluates both functional and aesthetic aspects. The functional component assesses the arc of motion in the sagittal plane, including flexion and extension, while the cosmetic component evaluates the carrying angle, representing coronal plane motion at the elbow joint. The Flynn criteria, as detailed in Table 3 below, indicate that a greater loss of motion in both sagittal and coronal planes corresponds to a poorer outcome. In addition to this, nerve injuries, specifically ulnar and radial nerve damage, were assessed in both groups to compare the incidence of nerve injury. Nerve injury was defined as any sensory or motor deficit, including weakness or numbness, attributed to damage to the ulnar or radial nerves.

**Table 3:** Flynn criteria

Outcome	Loss of carrying angle (cosmetic factor)	Loss of motion (functional factor)
Excellent	0°	0°
Good	≤ 5°	≤ 10°
Fair	6° to 10°	11° to 15°
Poor	> 10°	> 15°

Operative technique

**Figure 1:**
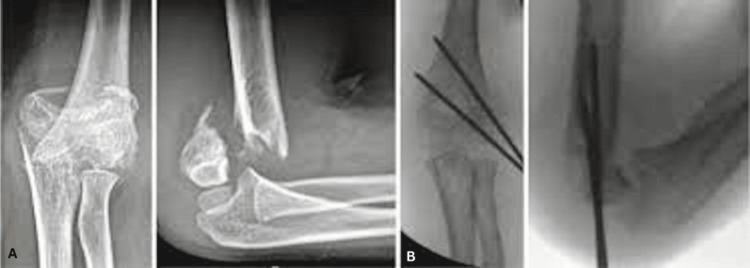
Divergent lateral pinning A: Preoperative anteroposterior and lateral radiographs of a 10-year-old boy; B: Immediate postoperative radiograph with good reduction

**Figure 2:**
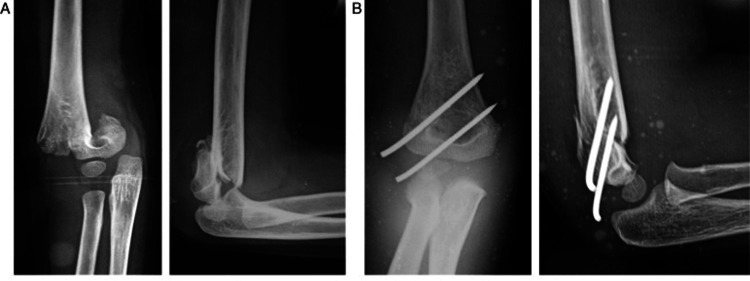
Parallel lateral K wires A: Preoperative anteroposterior and lateral radiographs of an eight-year-old boy; B: Immediate postoperative radiograph with good reduction

**Figure 3:**
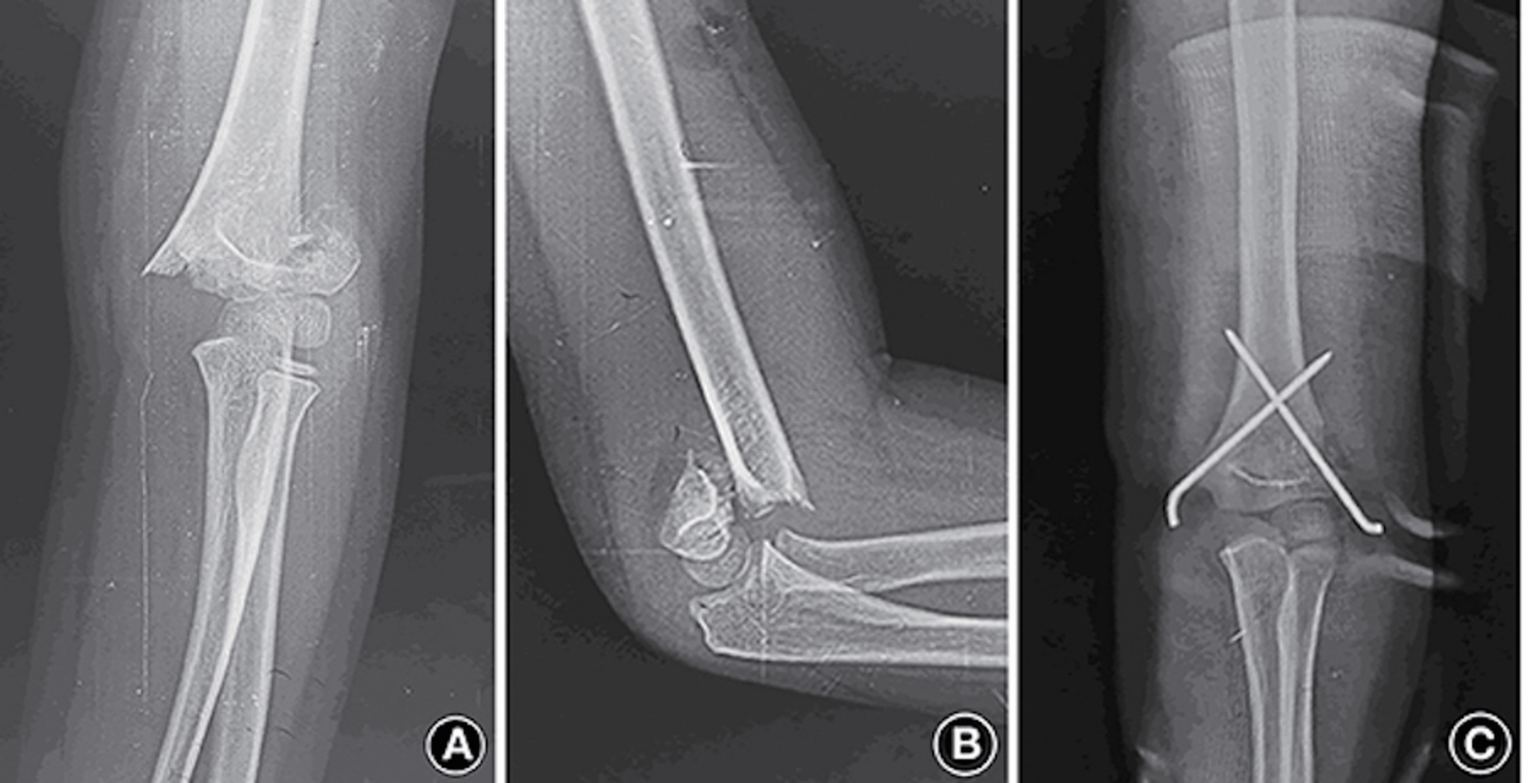
Type 3 SCFH treated with cross K-wire fixation A and B: Preoperative anteroposterior and lateral radiographs of an eight-year-old boy; C: Immediate postoperative radiograph with good reduction SCFH: Supracondylar fracture of the humerus, K-wire: Kirschner wire

**Figure 4:**
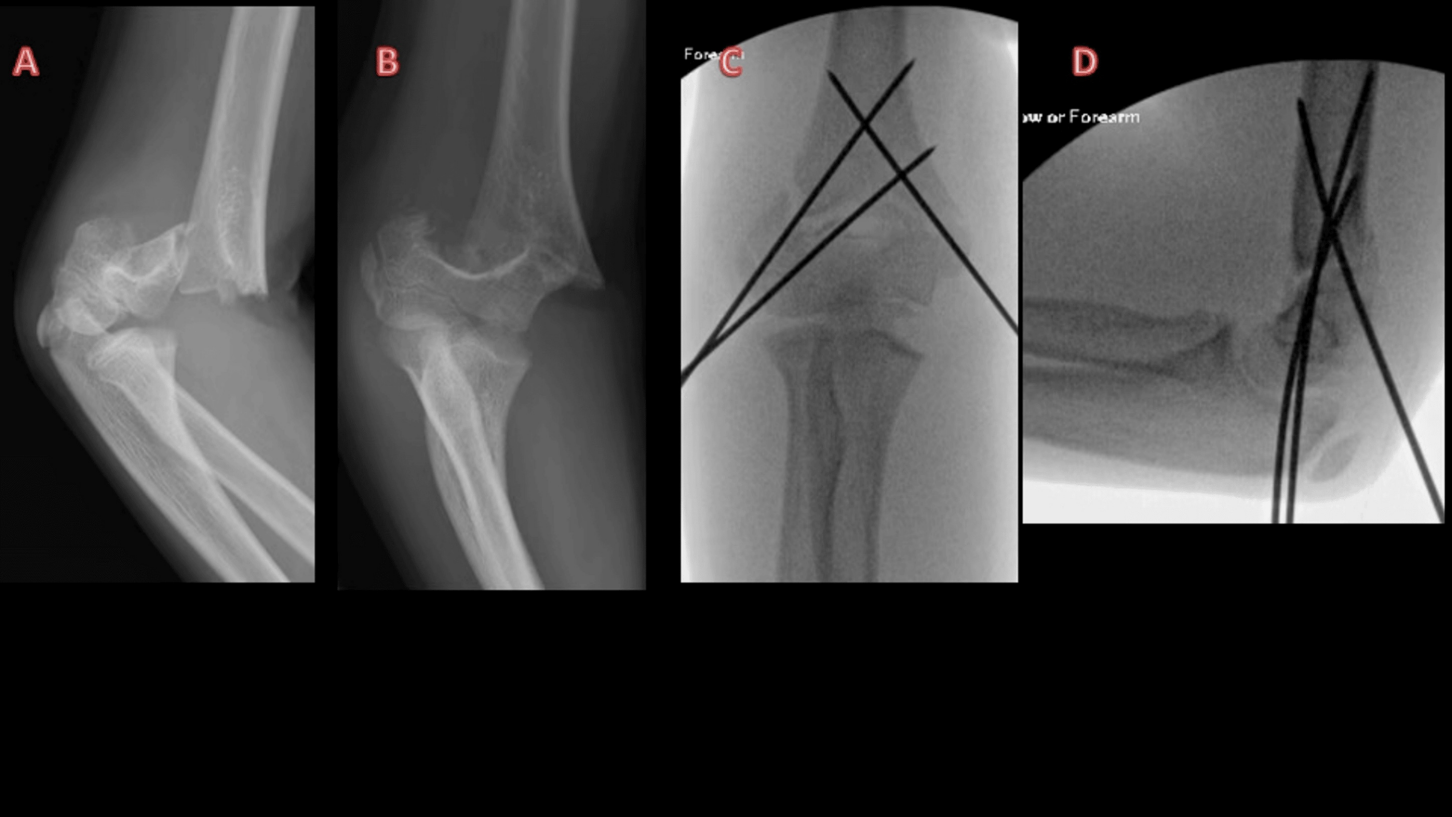
Type 3 supracondylar fracture treated with two lateral K wires and one medial K wire A and B: Elbow radiographs of a 10-year-old girl showing type 3 SCFH; C and D: Intraoperative radiographs of the same patient demonstrating reduction and fixation of fracture. Two Kirschner wires (K-wires) were used to stabilize the lateral column, and one K-wire was used to stabilize the medial column. SCFH: Supracondylar fracture of the humerus, K-wire: Kirschner wire

Under general anesthesia, patients were positioned supine with the injured limb off the table for manipulation and visualization under the C-arm for closed reduction and pinning. All fractures were manipulated preoperatively using a traction-countertraction technique, holding the elbow in hyperflexion with forearm pronation. Under C-arm guidance, two 1.6 mm or 2 mm K-wires were used, either laterally or across. To avoid ulnar nerve injury, a small skin incision was made medially, and the wire was inserted as anteriorly as possible with the elbow in slight extension during insertion for cross pinning. After achieving successful reduction, the fracture was stabilized with K-wires that were bent and placed just beneath the skin. A long arm splint was applied with the elbow positioned at 90° of flexion. On the second postoperative day, patients were discharged after postoperative radiographs (Figures 1-4) were taken to assess alignment and neurovascular function, and the dressing was checked. The cast, along with the K-wires, was kept in place for three weeks. After three weeks, follow-up X-rays were performed, and both the cast and K-wires were removed. This process was retrospectively reviewed for all patients included in the study.

## Results

The mean age of patients was 7.28 ± 2.03 years in group L and 8.20 ± 2.21 years in group C (p=0.10). In group L, there were 23 (41.81%) females and 32 (58.19%) males, while group C had 18 (32.72%) females and 37 (67.28%) males. The left side was more commonly affected, with 29 (52.72%) patients in group L and 32 (58.18%) patients in group C. Among the 110 patients in the study, 42 (76.36%) in group L and 46 (83.64%) in group C sustained injuries while playing. Road traffic accidents were the cause of eight (14.55%) patients in group L and six (10.91%) in group C, while falls from height accounted for five (9.09%) in group L and three (5.45%) in group C.

According to the Gartland classification, 44 (80%) of group L patients and 46 (83.64%) of group C patients had type IIIA fractures, while 11 (20%) of group L patients and nine (16.36%) of group C patients had type IIIB fractures. The mean surgical time was 26.2 ± 1.5 minutes for patients in group L and 29 ± 2.7 minutes for patients in group C, which was statistically significant (p=0.03). The mean follow-up period was 21.3 ± 1.4 weeks for group L and 23.5 ± 0.2 weeks for group C (p=0.33). Postoperative ulnar nerve injury was reported in six (5.45%) patients in group C, whereas no ulnar nerve injuries were reported in group L. Nerve injury was defined as the presence of sensory or motor deficits, including weakness, numbness, or impaired function of the affected nerve, specifically targeting the ulnar or radial nerves. A superficial infection was diagnosed in two patients (3.64%) in group C and four (7.27%) in group L, based on clinical signs such as localized redness, swelling, and discharge at the site. Pin loosening was observed in two patients (3.64%) in group C, with a diagnosis made through radiographic evaluation and physical examination, indicating abnormal movement at the pin site. No cases of pin loosening were reported in group L (0%).

A poor functional outcome, as defined by the Flynn criteria, is characterized by significant limitations in elbow mobility, noticeable deformities such as cubitus varus, and poor radiographic findings. Radiographic parameters, including the varus angle, play a crucial role in assessing these outcomes. A substantial reduction or reversal of the carrying angle, leading to a negative or abnormal angle, is a hallmark of cubitus varus, also known as a 'gunstock deformity.' This deformity is often an indicator of malunion, typically resulting from a loss of reduction or improper pin placement. Other radiographic markers, such as residual angular deformities or incomplete fracture healing, can further restrict elbow motion and contribute to unsatisfactory aesthetic results, which are integral to the Flynn criteria scoring. Therefore, both clinical and radiographic evaluations are vital for assessing functional recovery and identifying poor outcomes in patients with SCFH. All the aforementioned results are explained in Table 4.

**Table 4:** Demographic and clinical features of patients The asterisk (*) indicates a statistically significant difference between group L and group C (p<0.05).

Parameter	Group L	Group C	p-value
Age (years)	7.28 ± 2.03	8.20 ± 2.21	0.10
Gender			0.215
Male	32 (58.1%)	37 (67.2%)	
Female	23 (41.81%)	18 (32.72%)	
Sidedness			0.573
Left	29 (52.72%)	32 (58.18%)	
Right	26 (47.28%)	23 (41.82%)	
Mode of injury			0.616
Playing	42 (76.36%)	46 (83.64%)	
Road Traffic Accident	8 (14.55%)	6 (10.91%)	
Fall from height	5 (9.09%)	3 (5.45%)	
Type of fracture			0.403
III A	44 (80%)	46 (83.64%)	
III B	11 (20%)	9 (16.35%)	
Follow-up (in weeks)	21.3 ± 1.4	23.5 ± 0.2	0.33
Duration of surgery(minutes)	26.2 ± 1.5	29 ± 2.7	0.03*
Nerve injury	0 (0%)	6 (5.45%)	0.03*
Other complications			
Superficial infection	2 (3.64%)	4 (7.27%)	0.219
Pin loosening	2 (3.64%)	0 (0%)	0.154

According to the Flynn criteria, in group L, 44 patients (80%) had an excellent outcome, 10 patients (18.18%) had a good outcome, and one patient (1.82%) had a satisfactory outcome. In group C, 42 patients (76.64%) had an excellent outcome, nine patients (16.36%) had a good outcome, two patients (3.64%) had a satisfactory outcome, and two patients (3.64%) had a poor outcome in terms of functional outcome per the Flynn criteria. The results presented above are described in Table 5.

**Table 5:** Functional outcome (Flynn criteria)

Functional outcome	Group L	Group C	p-value
Excellent	44 (80%)	42 (76.64%)	0.387
Good	10 (18.18%)	9 (16.36%)	
Satisfactory	1 (1.82%)	2 (3.64%)	
Unsatisfactory	0 (0%)	2 (3.64%)	

## Discussion

Supracondylar humerus fractures are the most common pediatric elbow injury, accounting for more than 75% of all elbow fractures, with the highest incidence occurring between the ages of five and 11 years [13]. The primary goal of treatment for SCFH is to prevent varus and rotational deformities. Treatment options vary from conservative to surgical approaches, with non-displaced fractures typically managed conservatively. However, Gartland type III supracondylar fractures are highly unstable and require adequate reduction and firm fixation to prevent such deformities [14].

This study examined the outcomes of cross pinning versus lateral pinning in the treatment of supracondylar fractures. A total of 110 patients were retrospectively evaluated in both groups. The study found a predominance of male patients, with most injuries affecting the dominant upper extremity. The mean age was 7.28±2.03 years in group L and 8.20±2.21 years in group C, consistent with other studies. For instance, Naik et al. [5] reported mean ages of 7.20±2.21 and 6.28±2.03 years in their groups. Similarly, other studies by Babal et al. [15] and Khademolhosseini et al. [16] have reported similar age distributions, left-sided predominance, and a higher incidence in males, which aligns with the findings of this study [17,18].

In this study, the technique of lateral pinning was further divided into two configurations: parallel and divergent. Divergent lateral pinning, in particular, has been shown to provide greater biomechanical stability compared to parallel pinning, as the wider spread of pins increases fracture fixation strength. This is especially important in Gartland type III fractures, where fracture instability is high, and sufficient stabilization is crucial to prevent postoperative deformities. Both techniques were employed based on the fracture pattern and surgeon preference.

One of the major concerns with cross pinning is the risk of nerve injury. Lyons et al. reported that 6% of 375 patients developed iatrogenic ulnar nerve palsy postoperatively [19]. In contrast, Skaggs et al. found no cases of ulnar nerve palsy or loss of reduction in 124 children treated with lateral-entry pins alone, noting that cases of neurapraxia generally resolved over time. In this study, 5.45% of group C patients experienced postoperative nerve injury. Importantly, the radial nerve was not at risk in any of the patients, regardless of the approach used, which is consistent with previous studies reporting minimal radial nerve involvement in such fractures. The incidence of ulnar nerve injury can potentially be reduced by placing the lateral pin with the elbow flexed at 45° to 50° rather than in the hyperflexed position [20]. While nerve injury remains a concern with cross-pinning, lateral pinning, whether parallel or divergent, offers a safer alternative with a lower risk of iatrogenic nerve damage.

Additionally, two patients in group L and four in group C developed superficial infections, which were successfully treated with oral antibiotics. There were no cases of deep-seated infection, and no revision surgeries were required. The study also noted a statistically significant difference in operative time, with group C requiring more time. However, the actual difference of 2.8 minutes is likely clinically insignificant, suggesting that while the statistical outcome is notable, the short-time variation may not have a meaningful impact on clinical practice.

The overall outcomes were evaluated using Flynn’s adjusted criteria, a stringent classification system where any cubitus varus deformity, regardless of elbow function, is considered a poor outcome. The treatment methods employed in this study achieved excellent or good outcomes for the majority of patients. While the experience level of the surgeons was not explicitly discussed, the consistently positive outcomes across our patient group suggest that the effectiveness of the treatment approach is not significantly dependent on the surgeon's level of expertise. This is consistent with findings from other studies, such as the one by Shafi-Ur-Rehman et al. [21], which indicate that the treatment approach can be effective regardless of the surgeon's experience level. Thus, our results support the safety and efficacy of both parallel and divergent lateral pinning approaches across various levels of surgical expertise.

Limitations

This study has several limitations that must be acknowledged. The retrospective design may introduce bias due to potential errors or incomplete data, which could affect the reliability of the findings. The study was conducted at a single center, limiting the generalizability of the results to other settings or populations. Although the study included 110 patients, a larger sample size might have provided more definitive conclusions, especially regarding rare complications such as nerve injury. The follow-up period may have been too short to fully assess long-term outcomes and delayed complications. While Flynn's criteria were used to evaluate outcomes, they may not capture all aspects of functional recovery, including patient-reported outcomes related to pain, activity limitations, or quality of life. The absence of randomization in the assignment of patients to treatment groups introduces the possibility of selection bias, and varying levels of surgical expertise among the performing surgeons may have influenced the outcomes. These limitations suggest caution in interpreting the study’s findings and highlight areas for further research to strengthen the evidence base.

## Conclusions

For type III SCFH, lateral pinning fixation has proven to be an effective alternative, showing excellent functional outcomes and minimizing the risk of iatrogenic nerve injury, particularly to the ulnar nerve. This method provides sufficient fracture stability and demonstrates a favorable safety profile, making it a valuable option in settings where nerve injury is a significant concern. However, while our findings suggest that lateral pinning is a reliable technique, it is important to acknowledge the limitations of this study, including its single-center design, small sample size, and lack of long-term follow-up. These factors limit the generalizability of the results. Further research, including larger, multicenter studies with extended follow-up, is needed to confirm the long-term efficacy and safety of this approach.
